# Neuropsychological assessment of aggressive offenders: a Delphi consensus study

**DOI:** 10.3389/fpsyg.2024.1328839

**Published:** 2024-02-23

**Authors:** Juliette C. Hutten, Joan E. van Horn, Sylco S. Hoppenbrouwers, Tim B. Ziermans, Hilde M. Geurts, The Forensic Neuropsychology Consortium, Hasanen Al-Taiar

**Affiliations:** ^1^De Waag (Outpatient Forensic Mental Health Clinic), Forensic Care Specialists, Utrecht, Netherlands; ^2^Brain and Cognition, Department of Psychology, Faculty of Social and Behavioral Sciences, University of Amsterdam, Amsterdam, Netherlands

**Keywords:** neuropsychological tests, forensic psychology, aggression, violence, Delphi technique, Research Domain Criteria (RDoC)

## Abstract

**Objective:**

This study explores the intricate relationship between cognitive functioning and aggression, with a specific focus on individuals prone to reactive or proactive aggression. The purpose of the study was to identify important neuropsychological constructs and suitable tests for comprehending and addressing aggression.

**Methods:**

An international panel of 32 forensic neuropsychology experts participated in this three-round Delphi study consisting of iterative online questionnaires. The experts rated the importance of constructs based on the Research Domain Criteria (RDoC) framework. Subsequently, they suggested tests that can be used to assess these constructs and rated their suitability.

**Results:**

The panel identified the RDoC domains Negative Valence Systems, Social Processes, Cognitive Systems and Positive Valence Systems as most important in understanding aggression. Notably, the results underscore the significance of Positive Valence Systems in proactive aggression and Negative Valence Systems in reactive aggression. The panel suggested a diverse array of 223 different tests, although they noted that not every RDoC construct can be effectively measured through a neuropsychological test. The added value of a multimodal assessment strategy is discussed.

**Conclusions:**

This research advances our understanding of the RDoC constructs related to aggression and provides valuable insights for assessment strategies. Rather than suggesting a fixed set of tests, our study takes a flexible approach by presenting a top-3 list for each construct. This approach allows for tailored assessment to meet specific clinical or research needs. An important limitation is the predominantly Dutch composition of the expert panel, despite extensive efforts to diversify.

## 1 Introduction

Aggressive offenses have far-reaching consequences for individuals and society, including financial strain on health and justice sectors, public safety issues, reduced quality of life for victims, their relatives, and the offenders (Patel and Taylor, 2012; Langton et al., 2014; Rivara et al., 2019). Neuropsychological profiling is an underused clinical tool to assess the complex web of risk factors for aggressive behavior. This is surprising as the intricate relationship between cognitive functioning and reactive and proactive aggression has been widely studied. Although empirical studies and systematic reviews have uncovered neurocognitive mechanisms underlying reactive and proactive aggression (Alcázar-Córcoles et al., 2010; Kuin et al., 2015; Van De Kant et al., 2020), expert knowledge on individual neuropsychological assessment has not been integrated into the current body of research. An overarching framework that bridges the gap between fundamental research and clinical experience is therefore much needed. In the current study, a panel of experts is asked (i) which Research Domain Criteria (RDoC) domains (Insel et al., 2010; further explained below) are important in explaining reactive vs. proactive aggression and, (ii) which neuropsychological tasks are suitable for assessing those domains.

A common definition of aggression is “behavior that is intended to harm another person who is motivated to avoid that harm” (Allen and Anderson, 2017). Notably, aggressive offenders make up a substantial proportion (up to 70%) of prisons, forensic hospitals and outpatient mental health facilities (McMurran et al., 2000; Völlm et al., 2018). There is a great need for research into the risk factors of aggressive behavior to help reduce recidivism (Smeijers, 2017; Wigham et al., 2022). One of the potential risk factors for aggression that warrants exploration is cognitive functioning, particularly through neuropsychological assessments. Cognitive limitations are more prevalent among offenders than in the general population (Ogilvie et al., 2011), particularly among aggressive offenders (Cruz et al., 2020). For example, research found a prevalence of clinically significant executive deficits (a subset of cognitive functions) in an offender population ranging from 5.2% to 27.2% (correctional offenders) and 9.5–35.7% (forensic psychiatric patients), compared to 2.5% in the general population (Shumlich et al., 2019). Furthermore, multiple factors can be at the root of cognitive limitations, including traumatic brain injury, substance abuse, and attention deficit hyperactivity disorder, all of which are more prevalent among offenders (Harris, 2006; Ginsberg et al., 2010; Farrer and Hedges, 2011; Frost et al., 2013; Fayyad et al., 2017; Hellenbach et al., 2017; Muñoz García-Largo et al., 2020; Matheson et al., 2022). As such, it is necessary to further highlight the role of cognitive factors in the context of offending behavior, and this study aims to do so by improving knowledge about neuropsychological assessment in forensic populations.

### 1.1 Reactive and proactive aggression

The term “aggression” refers to a spectrum of acts that range from shouting or pushing to aggravated assault or homicide. By the definition stated above, rape, sexual assault, and robbery would also be classified as aggressive offenses. As the literature shows, most offenders are generalists, meaning they commit more than one type of crime in their lives (Simon, 1997; Soothill et al., 2000; Sullivan et al., 2006). Therefore, we chose to include aggressive sexual- or property crimes while non-aggressive crimes such as fraud were outside the scope of this study. Understanding the different determinants of aggression has been a subject of interest in various fields such as psychology, criminology, and neuroscience since the mid-20th century. Several taxonomies have been proposed in the literature (Parrott and Giancola, 2007; Krahé, 2013), but there is no consensus yet about which categorization is most appropriate. The most well-known distinction is the reactive-proactive dichotomy, sometimes referred to as hostile-instrumental (Buss, 1961). Reactive aggression occurs in reaction to a provocation or frustration and is impulsive in nature. Proactive aggression on the other hand is generally goal-directed and premeditated. Both types of aggression can occur within an individual, and thus, the strict classification into one of these two categories has been disputed in the literature (Bushman and Anderson, 2001). Currently, a dimensional view of aggression is favored, acknowledging that individuals often exhibit varying degrees of both reactive and proactive aggression rather than rigidly categorizing them into distinct types. Interestingly, research on factors associated with or related to reactive and proactive aggression provides empirical support for the usefulness of the distinction. For example, reactive aggression has been linked to heightened emotional reactivity, impulsivity, verbal impairments and impairments in executive functioning, and hostile attribution bias. Proactive aggression on the other hand is linked to a lack of moral emotions, callous and unemotional traits, and low physiological arousal (Cima and Raine, 2009). To summarize, individuals can exhibit both types of aggression, with a tendency toward one type, reflecting a predominant behavioral disposition. As both types of aggression appear to be related to different constructs, the current study considers both types of aggression separately.

### 1.2 Research Domain Criteria (RDoC)

**Figure 1:**
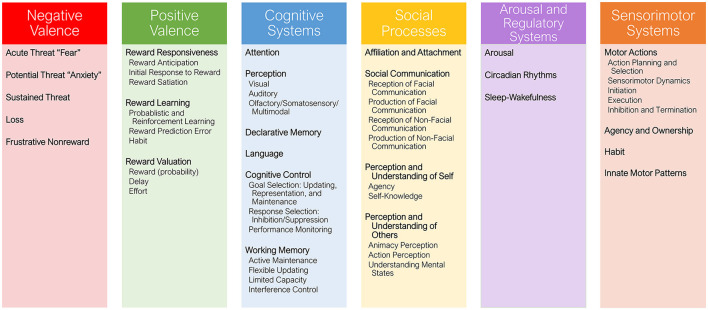
Overview of the Research Domain Criteria (RDoC) domains, including 25 constructs (bold) and 33 subconstructs (regular text).

The National Institute of Mental Health (NIMH) developed the RDoC (Insel et al., 2010), to—as opposed to traditional categorial diagnostic systems such as the Diagnostic and Statistical Manual of Mental Disorders (DSM; American Psychiatric Association, 2022)— investigate core dimensions of functioning that underlie various mental health conditions. In addition, as aggression can occur within various mental health conditions such as personality disorders, intermittent explosive disorder and conduct disorder, the RDoC framework provides a transdiagnostic perspective to uncover shared mechanisms that contribute to aggression across these diverse disorders. The RDoC describes six domains: (1) Negative Valence Systems, responsible for responses to aversive situations or context, such as fear, anxiety, and loss; (2) Positive Valence Systems, responsible for responses to positive motivational situations or contexts, such as reward seeking, consummatory behavior, and reward/habit learning; (3) Cognitive Systems, responsible for various cognitive processes; (4) Social Processes, which mediate responses to interpersonal settings of various types, including perception and interpretation of others' actions; (5) Sensorimotor Systems, responsible for the control and execution of motor behaviors, and their refinement during learning and development; and (6) Arousal/Regulatory Systems responsible for generating activation of neural systems as appropriate for various contexts, and providing appropriate homeostatic regulation of such systems as energy balance and sleep (see Figure 1).

### 1.3 Aggression and cognitive domains

In this section, the existing knowledge regarding the interplay between aggression and the domains outlined in the RDoC framework is briefly elucidated. If available, we refer to systematic reviews and/or meta-analyses. Neuropsychological studies have revealed differences between (aggressive) offenders and non-offending controls in different RDoC domains, such as Cognitive Systems (including executive functions, attention, and language) (Cohen et al., 2003; Ogilvie et al., 2011; Anderson et al., 2016; Burgess, 2020; Chow et al., 2022), Social Processes (Marsh and Blair, 2008; Karoglu et al., 2022), and Positive/Negative Valence systems (Estrada et al., 2019; Manning, 2020; mainly reward and threat processing). In our recent multi-level meta-analysis, we have studied all domains of cognitive functioning in relation to offending behavior (Hutten et al., preprint). Overall, offenders performed worse on neuropsychological tests than non-offending controls, and this was the case for all of the cognitive domains studied. A notable observation from this meta-analysis was the substantial variation in tests (146 different tests), and the lack of studies from non-Western countries. Through the Delphi method, we aim to gather insights from forensic neuropsychology experts across the world to obtain consensus on the most suitable tests to measure neuropsychological functioning in aggressive offenders. With this, we aim to expand on this empirical knowledge by connecting research findings and their translational application in forensic practice.

The primary goal of offender rehabilitation is reducing recidivism. A recent global systematic review found 2-year recidivism rates of 18–55% after incarceration and 10–47% after community sentences (Yukhnenko et al., 2023). Psychological treatment has a small but positive effect on recidivism in violent offenders, with a 10.2% difference in recidivism between treated vs. non-treated offenders (Papalia et al., 2019). Despite these findings, there remains a need for further enhancements in intervention strategies to reduce recidivism more effectively. More knowledge on the relation between the RDoC domains and aggression could enhance offender rehabilitation in several ways. Studies have found worse executive functioning in recidivists compared to first time offenders (Ross and Hoaken, 2011; Sánchez de Ribera et al., 2022). Conventional risk assessment tools appear to have reached their ceiling effect, achieving a moderate area under the curve of 0.70, (Monahan and Skeem, 2014; Ogonah et al., 2023). Risk-assessment tools often measure cognitive factors like impulsivity and self-control through less objective methods such as observer ratings and self-reports. Neuropsychological tasks are considered more objective, excluding the impact of compromised self-insight (Steward and Kretzmer, 2022). Accordingly, expanding risk assessment to include neuropsychological and neurobiological factors alongside the existing psychosocial risk factors may enhance the accuracy recidivism predictions (Aharoni et al., 2013, 2014; Haarsma et al., 2020; Zijlmans et al., 2021; Nauta-Jansen, 2022). In addition to predicting recidivism, cognitive functioning—in particular inhibitory and cognitive flexibility difficulties—also appears to predict treatment dropout and treatment success (Fishbein et al., 2009; Cornet et al., 2014). Identifying the specific cognitive domains that are impaired in offenders and related to aggression is crucial to providing targeted interventions and reducing the risk of criminal behavior. For example, an aggression regulation training could be suitable for individuals with aggression arising from inhibitory problems, while people with difficulties in emotion recognition might benefit more from an emotion recognition training (Li et al., 2023). Hence, misidentification of the determinants of the aggression may lead to suboptimal treatment.

Although a clear link has been demonstrated between cognitive limitations and aggression, the use of neuropsychology in forensic settings has not reached its full potential. For example, incorporation of neurobiological information in Dutch pretrial forensic reports was low and did not rise significantly from 2005 to 2015 (Kempes et al., 2019). Additionally, even when neurobiological factors were acknowledged in relation to the offense, they were often overlooked in discussions about future risk assessment and -management. There are three explanations for this observation which are not mutually exclusive. First of all, clinicians are likely to struggle identifying the most suitable instruments as there is a plethora of neuropsychological tests available. A systematic review on neuropsychological assessment practices in forensic settings found a notable diversity in assessment tools, with 140 different types of tests. The authors conclude that a wide range of neuropsychological functions are being measured by a large number of instruments (Venturi Da Silva and Cavalheiro Hamdan, 2022). Related to this, many tests have multiple outcomes—often measuring different cognitive functions—or multiple ways to calculate the outcomes. This heterogeneity may compromise the reliability of test results and raises questions about how information is understood by clinicians and legal practitioners (Serafim et al., 2015). Second, for most neuropsychological tests normative data are collected from general population samples and have not been validated for the offender population. Possibly, the use of default norm scores leads to insufficient differentiation among individuals in the offender setting (Cornet et al., 2016). As such, it remains unclear which tests are most sensitive and suitable for the aggressive offender population. Third, offender populations present unique challenges in conducting neuropsychological assessments, such as high rates of noncompliance, low motivation (for treatment and/or assessment), and limited education and literacy levels (Hetland et al., 2007; Tuominen et al., 2014). Cultural and linguistic differences may also need to be considered when conducting neuropsychological assessments with offender populations. Considering these challenges, further research and tailored approaches are required to address the selection of suitable tests and norms for the aggressive offender population, to ensure accurate and reliable assessments.

### 1.4 Study objectives

This study aims to identify the most suitable neuropsychological tests for cognitive assessment within the aggressive offender population, distinguishing between predominantly reactive vs. proactive aggressive offenders. With this, our research contributes to the advancement of forensic neuropsychology. By pinpointing the specific cognitive domains associated with both reactive and proactive aggression, we aim to pave the way for more targeted assessments and interventions in aggressive offender populations. To achieve this goal, we need to bridge the gap between research and clinical practice and strife toward consensus among an international panel of experts from the field of forensic neuropsychology. In the current study, we will apply the Delphi methodology for this purpose. Our objectives encompass two categories of questions posed to the expert panel: firstly, we seek theoretical insights into the constructs commonly associated with aggression, emphasizing their significance in the evaluation of aggressive offenders; secondly, we aim to pinpoint the most suitable tests for this evaluation, thereby facilitating future test selection in forensic contexts.

## 2 Materials and methods

This study was preregistered at AsPredicted (#103758) and has been approved by the Ethics Review Board (ERB) of the University of Amsterdam (ERB number: 2022-BC-15289).

### 2.1 The Delphi methodology

We conducted a Delphi consensus study to obtain consensus among an international panel of experts in forensic neuropsychology. While meta-analyses and reviews allow us to have and overview of the current scientific knowledge, the Delphi method allows us to obtain insight into the existing clinical expertise. The Delphi method is a technique used to achieve consensus among a group of experts by soliciting their opinions through a series of questionnaires and providing them with controlled feedback (Dalkey and Helmer, 1963). The Delphi method is based on the concept of collective wisdom, which assumes that the combined opinion of multiple people is closer to the truth than a single individual's perspective (Habibi et al., 2014). Recently, researchers have been striving to achieve consensus on various neuropsychological topics, such as the definition of the term ‘impairment' or inconsistent use of test score labels (e.g., Guilmette et al., 2020). Our study aligns with these developments. The Delphi methodology, with its collaborative and iterative nature, serves as an effective tool within this context, facilitating the establishment of a shared foundation for understanding and addressing diverse neuropsychological considerations in the field. This is carried out by aggregating the results of online, anonymous questionnaires in a systematic way. The current study consisted of three rounds, which are described in 2.4 Procedure and data analysis.

### 2.2 Expert panel selection

Both researchers and clinicians employed in the field of forensic neuropsychology were invited to participate in the panel. Potential researchers were identified based on the articles that emerged from our literature review which is in review (Hutten et al., preprint). The researchers who had a minimum of two publications on the topic of forensic neuropsychology, of which one in the last five years (to confirm that they were still actively engaged in the field) were approached to participate in the Delphi study. For clinicians, the inclusion criterium is at least 4 years' experience as a (clinical) neuropsychologist in the forensic setting. Recruitment took place through the author's networks, (international) societies or networks for neuropsychology/forensic psychology, social media, and through the “snowballing technique” (Iqbal and Pipon-Young, 2009). Panels with 10 to 50 members are recommended for Delphi studies (Turoff, 1975). In total, 127 potential experts were invited personally by email. Sixty-three potential experts started the questionnaire and provided digital informed consent. Thirty potential experts responded they could not participate (no time: 13, questioned their own expertise: 15, no reason: 2). Finally, 32 experts completed the first-round questionnaire.

### 2.3 Research Domain Criteria (RDoC)

This Delphi study was based on the RDoC framework (Insel et al., 2010). The RDoC model is a research framework that approaches mental health and psychopathology by examining major domains of basic human neurobehavioral functioning, rather than relying on traditional diagnostic categories. The model consists of six major functional domains (see Figure 1), and each domain is studied by exploring different aspects using constructs that are examined across a range of functioning from normal to abnormal.

### 2.4 Procedure and data analysis

In three consecutive rounds of online questionnaires (compiled through Qualtrics, 2023), experts rated the importance of a predetermined list of the RDoC constructs on a 5-point scale from 1 “not important” to 5 “essential”, with a non-neutral midpoint of 3 (moderately important). Using a non-neutral midpoint forces panelists to deliberate and to decide about the importance of the constructs. If they felt incompetent to answer a question, a “don't know” option was available (Linstone and Turoff, 1975). Subsequently, the panel members provided suggestions for tests that can be used to measure the constructs they rated at least moderately important (rating 3 or higher). In addition, they rated each other's test suggestions as suitable or not suitable for aggressive offenders. Throughout the questionnaires, panel members can provide explanations or reasoning. Before distributing the questionnaire for the first round, two clinical neuropsychologists filled in the questionnaire to provide feedback and ensure clarity of the questions.

After each round, the constructs that did not achieve consensus (about their importance) moved into the subsequent round for re-rating. Our operationalization of consensus is interquartile range (IQR) ≤ 1. For a four to five-point Likert scale, an IQR of 1 or less is considered a high level of consensus (Raskin, 1994; Rayens and Hahn, 2000).

For the importance ratings of the RDoC constructs, means and standard deviations are reported. We conducted Mann-Whitney U tests to analyze the difference in importance scores between reactive and proactive aggression, primarily due to the ordinal nature of the data. For the suitability of tests, we reported the percentage of the panel that rated the test as suitable.

#### 2.4.1 Round 1

The objectives of the first round were (1) to identify the most important RDoC constructs that should be included in the assessment of aggressive offenders, and (2) to collect suggestions for tests that are recommended to assess these constructs. Before the experts started with the main questions, they were asked to fill in some information about their age, gender, profession, current workplace, and academic degree.

Then, the panel members were asked to rate the importance of the RDoC constructs. For the constructs they rated as at least moderately important, they were also asked to rate the importance of the underlying subconstructs. The experts were able to suggest additional constructs not delineated in the RDoC. The research team (JH, JvH, SH, TZ, and HG) evaluated these suggestions to confirm they were not already covered in the RDoC, they were clearly described, and they were within the scope of the RDoC [as suggested by Jorm (2015)]. These additional constructs were then added to subsequent rounds.

Next, for the constructs that they rated as at least moderately important, the experts gave suggestions for tests that they recommend for the assessment of this construct. They could give several suggestions per construct.

#### 2.4.2 Round 2

The 32 panel members who completed round 1 were invited to participate in round 2 of the study, which 26 of them did. (Sub)constructs that did not reach consensus in round 1 were rated again. These constructs were presented along with feedback outlining the average panel rating, each expert's own previous response, and a synopsis of comments that were offered by experts in support of their opinion. In addition, the additional constructs added by the panelists in round 1 were rated for importance. Then, the experts scored the suitability of the recommended tests suggested in round 1 (suitable/not suitable/don't know). If the round 1 tests suggestions were not specific enough—e.g., a test category such as “gambling tests” or a measurement goal such as “verbal comprehension tests”—the panel was asked to specify in this round.

#### 2.4.3 Round 3

The 26 panel members who completed round 2 were invited to participate in round 3 of the study. Round 3 was completed by 24 panel members. This round was mostly similar to round 2. In addition, the top-3 tests that were rated most frequently as suitable and were known by at least half of the panel were presented to the panel members. They were asked to rank these tests from most to least suitable.

## 3 Results

Thirty-two experts completed round 1 of the study (mean age = 43.44, SD = 11.20, 15 males, 17 females). Characteristics of the expert panel are displayed in Table 1. Despite repeated attempts (see paragraph 2.2) to gather an international expert panel, most experts were currently working/living in the Netherlands (*n* = 17). Seven of the experts were researchers, nine were clinicians, fifteen professionals integrated their therapeutic work with scientific research, and one was currently employed as manager. Of the original panel, twenty-four completed all three rounds of questionnaires and were included in the consortium.

**Table 1:** Characteristics of the initial expert panel (*N* = 32).

**Demographics**	
Gender, male/female *N*	15/17
**Age M (SD)**	43.4 (10.7)
**Profession**^a^ ***N*** **(%)**	
**Researcher**	22 (68.8)
**Clinician**	24 (75.0)
**Other (e.g., teaching, management)**	8 (25.0)
**Country**, ***N***(**%**)	
**Netherlands**	17 (53.1)
**USA**	5 (15.6)
**Italy**	5 (15.6)
**India**	2 (6.3)
**UK**	1 (3.1)
**Australia**	1 (3.1)
**Sweden**	1 (3.1)
**Setting**^a^ ***N*** **(%)**	
**University**	13 (40.1)
**Outpatient/ambulatory**	12 (37.5)
**(Forensic) hospital/inpatient**	12 (37.5)
**Diagnostics/Assessment**	5 (15.6)
**Research center**	4 (12.5)
**Prison/correctional facility**	2 (6.25)
**Assisted living**	1 (3.1)
**Academic rank**^b^ ***N*** **(%)**	
**Professor**	5 (15.6)
**Associate professor**	3 (9.4)
**Assistant professor**	4 (12.5)
**Post-doctoral researcher**	5 (15.6)
**PhD candidate**	2 (6.3)
**Master of Science**	3 (9.4)
**Type of clinician**^c^ ***N*** **(%)**	
**Clinical neuropsychologist**	10 (31.3)
**Neuropsychologist**	5 (15.6)
**Clinical psychologist**	3 (9.4)
**Psychiatrist**	2 (6.3)
**Neurologist**	1 (3.1)
**Other**	3 (9.4)
**Years of experience in the forensic setting**^c^ ***N*** **(%)**	
**< 4**	5 (15.6)
**4–7**	7 (21.9)
**8–11**	5 (15.6)
**12–15**	2 (6.3)
**16+**	5 (16.1)

^a^Panel members could give multiple answers to this question. ^b^This question was only answered for the panel members who were employed as researcher. ^c^This question was only answered for the panel members who were employed as clinician.

In round 1, for each construct a panel member rated as at least moderately important (rating 3 or higher), the panel member was asked to suggest one or more tests to measure this construct. In total, 223 different tests were suggested by the panel.

In round 2, the panel rated these tests as “suitable”, “not suitable” or “don't know”. In round 3, we presented the panel with the three most-suitable tests (that were known by at least half of the panel) per construct, and we asked them whether they agreed with this top three. However, many did not fill in these questions. One explanation is that they did not know one or more of the tests, making it impossible to rank them. Another possibility is a decrease in motivation as the questionnaires were quite extensive and time consuming. Because of this, we based the top-3 tests in Table 3 on the suitability ratings from round 2. For certain constructs, fewer than three tests were familiar to at least half of the panel, resulting in less than three test suggestions (or even zero) being included in the overview.

To aid clinicians in their test selection, we included some practical information about the administration time, age range, manual, and psychometric properties of the tests. We derived this information from test manuals, systematic reviews, and books. If these were not available, we reported on single studies with a sample that was most similar to the aggressive offender population. Our goal was not to create an exhaustive and comprehensive overview, as it falls beyond the scope of this study. Therefore, we refer readers to the British Psychological Society test reviews using the EFPA review model (British Psychological Society, n.d.), the Buros Center for Testing (Buros Center for Testing, n.d.), or for Dutch readers the COTAN (NIP, n.d.) for more information about the psychometric properties of tests.

**Figure 2:**
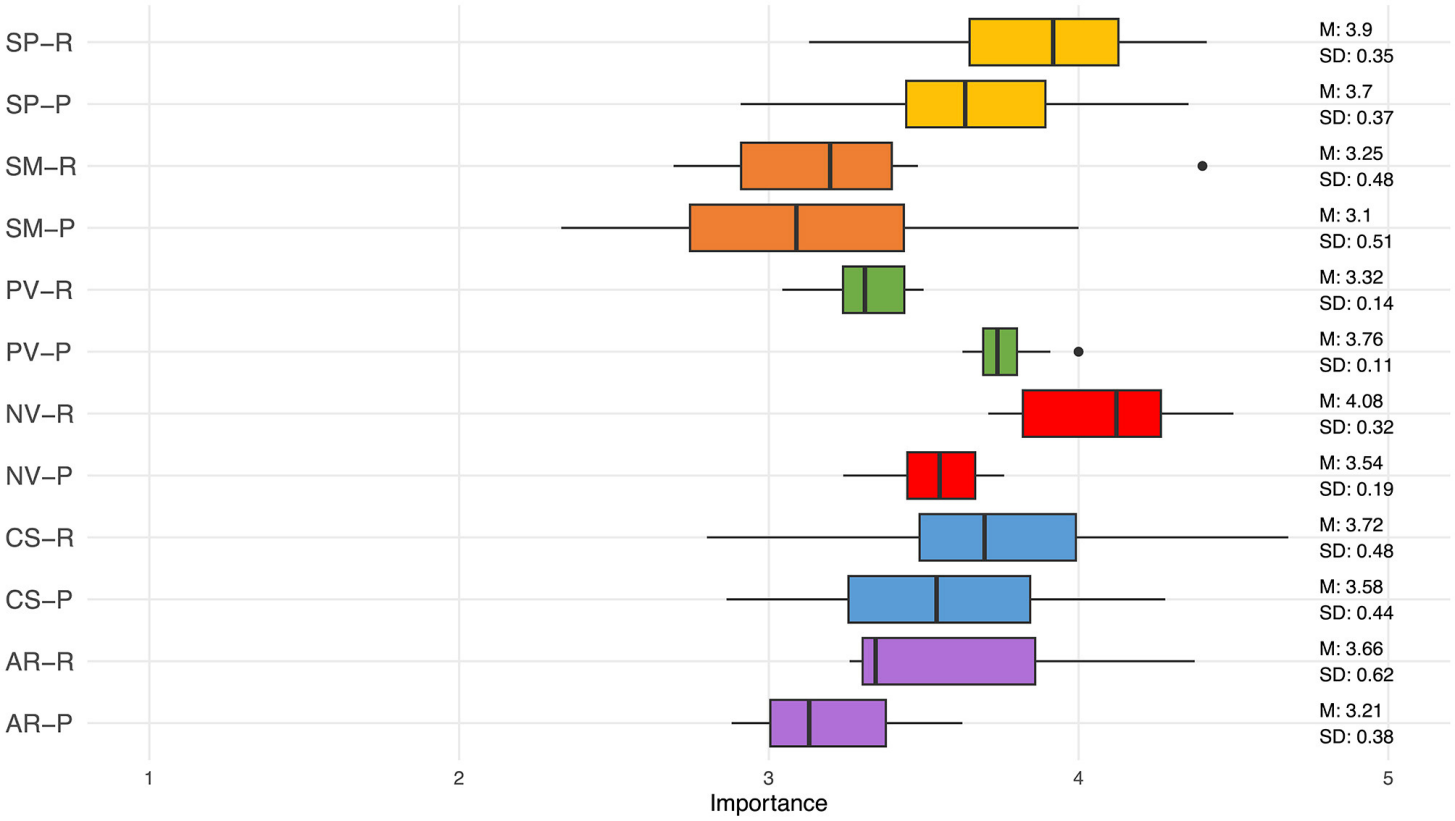
Boxplots of importance scores of the RDoC domains for reactive (R) and proactive (P) aggression. SP, Social Processes; SM, Sensorimotor Systems; PV, Positive Valence Systems; NV, Negative Valence Systems; CS, Cognitive Systems; AR, Arousal/Regulatory Systems.

Below, we discuss the results per domain, sorted by importance-rating (see Table 2 and Figure 2). First, the importance ratings are discussed, including the reasoning provided by the panel members. Then, the test suggestions are discussed.

**Table 2:** Final ratings of the RDoC constructs.

	**Reactive**					**Proactive**				
**RDoC constructs**	***n***	**Range**	**M**	**SD**	**IQR**	***n***	**Range**	**M**	**SD**	**IQR**
**Cognitive Systems**			3.72	0.48	1			3.58	0.44	1
Attention	26	1–5	3.65	0.80	1	24	1–5	3.58	0.88	1
Perception	23	1–4	3.30	0.76	1	22	1–4	3.23	0.87	1
Visual	23	2–5	4.00	0.74	0	23	2–5	3.35	0.83	1
Auditory	23	1–5	3.74	0.96	1	22	1–5	3.05	0.90	0
Olfactory/somatosensory/multimodal	24	1–4	3.00	0.72	0	22	1–4	2.86	0.89	1
Declarative memory	25	1–4	2.80	0.71	0	24	1–5	3.13	0.85	1
Language	26	1–4	3.19	0.69	0	24	1–5	3.08	0.83	1
Cognitive control	31	2–5	4.45	0.72	1	31	2–5	4.16	0.93	1
Goal selection, updating	30	2–5	4.03	0.89	1	31	3–5	4.26	0.68	1
Representation, and maintenance response selection	31	3–5	4.68	0.60	1	25	3–5	4.28	0.54	1
Inhibition/suppression performance monitoring	30	3–5	4.23	0.68	1	30	2–5	4.17	0.79	1
Working memory	31	1–5	3.45	1.15	1	30	1–5	3.37	1.19	1
Active maintenance	27	1–5	3.59	1.08	1	24	2–5	3.67	0.70	1
Flexible updating	29	2–5	3.97	0.91	1	24	1–5	3.75	0.90	0
Capacity	25	1–5	3.64	0.86	0	28	1–5	3.50	1.11	1
Interference control	25	2–5	3.92	0.64	1	24	2–5	3.88	0.74	1
Counterfactual reasoning^*^	20	2–5	3.75	0.79	1	19	2–5	3.68	0.75	1
Information processing speed^*^	25	2–5	3.64	0.64	1	24	2–5	3.50	0.72	1
**Arousal/regulatory**			3.66	0.63	0			3.21	0.38	0
Arousal	32	1–5	4.38	1.10	1	24	2–5	3.63	0.65	1
Circadian rhythms	23	2–4	3.26	0.54	1	24	2–4	2.88	0.54	0
Sleep-wakefulness	29	1–5	3.34	1.20	1	23	2–4	3.13	0.76	1
**Negative valence systems**			4.08	0.32	1			3.54	0.19	0
Acute threat “fear”	32	1–5	4.50	0.84	1	25	1–5	3.24	0.83	1
Potential threat “anxiety”	32	3–5	4.31	0.69	1	32	1–5	3.44	1.05	1
Sustained threat	25	3–5	4.12	0.60	1	25	1–5	3.68	0.80	1
Loss	25	1–5	3.72	0.84	0	23	1–5	3.48	0.85	1
Frustrative nonreward	32	1–5	4.13	0.94	1	25	1–5	3.76	0.72	0
The ability to learn from one's own errors^*^	24	3–5	3.71	0.55	1	24	2–4	3.63	0.58	1
**Positive valence systems**			3.32	0.14	0			3.76	0.11	0
Reward responsiveness	23	2–4	3.26	0.62	1	22	2–4	3.64	0.58	1
Reward anticipation	28	1–5	3.46	1.10	1	21	3–4	3.76	0.44	1
Initial response to reward	28	1–5	3.39	1.13	1	23	2–5	3.70	0.63	1
Reward satiation	23	2–4	3.04	0.64	0	22	2–5	3.68	0.65	1
Reward learning	22	3–4	3.50	0.51	1	21	3–5	3.76	0.54	1
Probabilistic and reinforcement learning	21	2–4	3.48	0.60	1	22	3–5	3.91	0.43	0
Reward prediction error	21	2–4	3.43	0.60	1	22	3–5	4.00	0.53	0
Habit - pvs	24	2–4	3.21	0.59	1	24	2–5	3.63	1.01	1
Reward valuation	22	2–4	3.18	0.73	1	21	3–4	3.86	0.36	0
Reward (probability)	23	2–4	3.35	0.65	1	23	2–5	3.78	0.60	1
Delay	22	2–4	3.27	0.63	1	21	2–5	3.71	0.72	1
Effort	20	2–4	3.25	0.64	1	20	2–5	3.70	0.73	1
**Sensorimotor**			3.25	0.48	1			3.10	0.51	1
Motor actions	28	1–5	3.21	1.03	1	22	2–4	2.72	0.55	1
Action planning and selection	22	2–4	3.18	0.59	1	22	2–5	3.45	0.80	1
Sensorimotor dynamics	22	1–4	2.86	0.56	0	24	1–5	2.94	0.85	0
Initiation	22	1–4	3.36	0.73	1	24	1–5	3.38	1.09	1
Execution	22	1–5	3.41	0.85	1	24	1–5	3.56	1.09	1
Inhibition and termination	20	2–5	4.40	0.82	1	24	2–5	4.00	0.82	1
Agency and ownership	27	1–5	3.48	1.01	1	21	1–5	3.24	0.94	1
Habit – sensorimotor	26	1–5	2.81	1.06	1	21	1–3	2.33	0.58	1
Innate motor patterns	26	1–5	2.69	1.16	1	20	1–4	2.60	0.75	1
Sensorimotor integration^*^	16	1–4	3.06	0.85	1	17	1–4	2.82	0.81	1
**Social processes**			3.90	0.35	1			3.70	0.37	1
Affiliation and attachment	23	1–5	3.74	0.81	1	31	2–5	4.19	0.83	1
Social communication	23	3–5	4.00	0.67	1	21	3–5	3.86	0.48	1
Reception of facial communication	29	3–5	4.38	0.68	1	22	3–4	3.68	0.48	1
Production of facial communication	23	3–4	3.57	0.51	1	21	2–4	3.33	0.66	1
Reception of non-facial communication	29	4–5	4.41	0.50	1	21	3–4	3.57	0.51	1
Production of non-facial communication	23	2–5	3.52	0.67	1	28	1–5	3.39	1.20	1
Perception and understanding of self	32	2–5	4.16	0.77	1	31	2–5	4.19	0.75	1
Agency	30	1–5	3.97	1.03	1	21	3–5	3.86	0.57	1
Self-knowledge	30	1–5	3.73	1.11	1	29	1–5	3.59	1.05	1
Perception and understanding of others	32	1–5	4.25	0.95	1	31	3–5	4.35	0.66	1
Animacy perception	23	2–5	3.87	0.55	0	27	1–5	3.41	1.12	1
Action perception	29	1–5	4.00	1.04	1	29	1–5	3.55	1.06	1
Understanding mental states	31	3–5	4.35	0.71	1	31	2–5	4.06	0.77	1
Ability to correctly understand the authenticity of others emotions^*^	22	3–5	4.05	0.49	0	19	2–5	3.79	0.71	1
Ability to understand absurdities^*^	23	2–5	3.13	0.69	0	22	1–4	2.91	0.61	0
Emotional contagion^*^	19	2–5	3.53	0.84	1	18	2–5	3.33	0.84	1
Moral reasoning^*^	23	2–5	3.87	0.97	1	21	1–5	3.90	1.00	1
Sympathy^*^	21	2–5	3.62	0.81	1	21	1–5	3.57	0.98	1
**Other**										
Intelligence/IQ^*^	24	2–5	3.54	0.83	1	24	2–5	3.75	0.79	1
Symptom/performance validity^*^	21	1–5	2.90	1.22	2	21	1–5	3.14	0.12	2
Cognitive distortions^*^	23	2–5	3.78	0.74	1	23	2–5	3.87	0.87	1
Emotion regulation^*^	23	2–5	4.39	0.84	1	23	2–5	3.74	1.01	2

^*^These constructs are not part of the RDoC, but were suggested as additions in round 1.

### 3.1 Negative valence systems

Negative Valence Systems were rated as the most important domain (M = 3.81, SD = 0.14), with a significant difference between reactive (M = 4.08, SD = 0.32) and proactive (M = 3.54, SD = 0.19) aggression (*U* = 2, *p* = 0.009). The ability to learn from one's own errors was suggested as an addition to this domain in round 1. Reaction to threat was rated as more important for reactive than for proactive aggression (acute: M = 4.50 vs. 3.24, potential: M = 4.31 vs. 3.44, sustained: M = 4.12 vs 3.68). The panel reasoned that as reactive aggression is driven by an immediate emotional reaction to a perceived threat or provocation, these constructs are more relevant in reactive aggression. Anxiety might make individuals more sensitive to perceived provocations, increasing the likelihood of aggression. Prolonged exposure to threat (sustained threat) might result in chronic stress and might cause individuals to use aggression to end the threat. Loss and being unable to achieve goals or experience rewards (frustrative non-reward) can lead to feelings of anger, sadness and disappointment. Aggression might be a way to cope with these feelings.

In total, 22 neuropsychological tests were suggested by the panel to assess Negative Valence Systems. The panel commented that it might be better to assess this domain by including biological measures (heart rate, eye tracking/pupil size, skin conductance) or self-report. For frustration, observation from potentially frustrating tests was also suggested, however, it was noted that test observations should not be confused with objective test results. Frustration from not performing the test correctly is not the intended measurement of the test and therefore, not an objective test result. For some Negative Valence constructs, it was impossible to validly assemble a top-3 as many tests that were suggested were unknown by more than half of the panel. Therefore, Acute Threat and Loss have only two tests in the overview and Sustained threat only one.

### 3.2 Social processes

The domain Social Processes was rated as the second most important overall (M = 3.80, SD = 0.33), with a nonsignificant difference between reactive (M = 3.90, SD = 0.35) and proactive (M = 3.70, SD = 0.37) and aggression (*U* = 190.5, *p* = 0.097). Four additional constructs were added to this domain: sympathy, moral reasoning, ability to correctly understand the authenticity of others' emotions, and emotional contagion. The panel commented that the ability to interpret social cues (including other people's emotions and social ambiguity), is crucial in understanding and preventing aggression. Misinterpretations can increase the risk of both reactive and proactive aggression. However, proactive aggression may be less influenced by this as people with high callous-unemotional traits tend to be less concerned with other people's emotions. Another important aspect within this domain is empathy. High callous unemotional traits in individuals displaying proactive aggression often involves cognitive empathy without affective empathy, enabling manipulative behavior.

It was noted by the panel that it might be more feasible to assess Social Processes with interviews, questionnaires and observations instead of neuropsychological tests. In total, 34 tests were suggested for this domain, resulting in a top-3 tests for each construct.

### 3.3 Cognitive systems

Next, Cognitive Systems were rated as M = 3.65 (SD = 0.44), with a non-significant difference between reactive (M = 3.72, SD = 0.48) and proactive aggression (M = 3.58, SD = 0.44; *U* = 134.5, *p* = 0.389). The panel reflected on why the Cognitive Systems are (not) important to consider in aggressive patients. Working memory is needed to process and react to triggers (reactive aggression), but on the other hand, working memory is required to plan proactive aggressive behaviors. Attention was deemed important in reactive aggression, as it can be biased toward potential threats, while ignoring neutral or friendly information. Cognitive control is linked to inhibition, which can help prevent future (especially reactive) aggression. In addition, cognitive control is important to be able to find non-aggressive solutions to problems, and to apply lessons from therapy into daily life (also related to declarative memory). Language was considered important in assessing aggression because poor verbal skills can hinder the ability to find non-aggressive solutions in conflicts, potentially leading to misunderstandings and frustration. The role of perception was somewhat unclear among the panel members. Counterfactual reasoning and information processing speed were added as additions to this domain.

For Cognitive Systems, a large number (154) of different tests was suggested. The top-3 tests per construct are displayed in Table 3.

**Table 3:** Characteristics of the top-3 most suitable tests per construct, sorted by highest importance rating (*N* = 25).

**Construct (importance rating: reactive, proactive) tests (authors)**	**Suitable (%)**	**Known by (%)**	**Short description**	**Administration time (min)**	**Age**	**Psychometric properties**
**Cognitive control (R: 4.45, P: 4.16)**						
Go/No Go task^*^	84%	84%	Computerized task that measures the ability to stop automatic reactions (impulse control).	Varies	18-65	Convergence with other types of self-control measures (Duckworth and Kern, 2011) • Executive functions: *r* = 0.16, N = 4855 • Delay tasks: *r* = 0.12, N = 523 • Self-report: *r* = 0.11, N = 1969 • Informant-report: *r* = 0.15, N = 1883
Wisconsin Card Sorting Task (WCST) (Heaton et al., 1993)^*^	84%	88%	Sorting cards based on changing rules and adapting. Measures ability to shift strategies, problem-solve, and assesses frontal lobe function.	20-30	5-89	Strauss et al. (2006) • Test-retest: generally low • Evidence of sensitivity to frontal damage: yes – but poor sensitivity and specificity • Evidence of ecological ability: yes
D-KEFS: Color Word Interference test (CWIT) (Delis et al., 2001)	80%	84%	Assesses cognitive functions like inhibiting automatic responses and shifting. Naming the ink color of words while inhibiting reading.	10	8-89	Internal consistency • Adequate (0.70–0.79) (Strauss et al., 2006), 0.75 (combined naming + reading) (Delis et al., 2007a) Test-retest • Marginal (0.60–0.69) to adequate (0.70–0.79) (Strauss et al., 2006) • Test-retest: 0.74 (cond. 1), 0.61 (cond. 2), 0.72 (cond. 3), 0.64 (cond. 4) (Delis et al., 2007a)
**Perception and understanding of others (R: 4.25, P: 4.35)**						
Faux Pas test (Stone et al., 1998; Gregory et al., 2002)	83%	83%	Measures detection social blunders in conversations through a number of stories.	15-20	18-65	(Söderstrand and Almkvist (2012) • Internal consistency: 0.905 • Split-half: 0.954 • Interrater: 0.916 • correlated significantly with the Eyes Test (*r* = 0.302, *p* ≤ 0.05) and the Dewey Story Test (*r* = −0.276, *p* ≤ 0.05) Osterhaus and Bosacki (2022) • Internal consistency: reported by 3 studies (0.78, 0.81, 0.91; M = 0.83, SD = 0.07)
Emotion Recognition Task (ERT) (Montagne et al., 2007)^*^	78%	78%	Computer-generated paradigm designed to assess the recognition of 6 basic facial emotional expressions: anger, disgust, fear, happiness, sadness, and surprise.	6-10	8-75	• Studies demonstrated the validity in a wide range of patient groups, often showing impairments that are selective for specific emotions (see Kessels et al., 2014 for an overview)
Strange stories (Happé, 1994)	70%	74%	Gauges social understanding. Participants interpret social scenarios, assessing comprehension of subtle social cues.	30-45 (original version) 15-20 (SS-R)	Developed for children, but can also be used for adults	Osterhaus and Bosacki (2022) • Internal consistency: reported by 5 studies (0.67, 0.69, 0.69, 0.73, and 0.79; M = 0.71, SD = 0.04)
**Perception and understanding of self (R: 4.16, P: 4.19)**						
Thematic Apperception Test (TAT) (Morgan and Murray, 1935)	52%	65%	Creating stories based on ambiguous pictures, revealing inner perceptions and imagination.	2 sessions of 50 min.	5+	Hilsenroth and Segal (2004) • Interrater reliability: 0.80−0.86
Emotion Recognition Task (ERT)	48%	70%	See above			
Faux pas test	43%	74%	See above			
Strange stories test	43%	70%	See above			
**Arousal (R: 4.38, P: 3.63)**						
Go/No Go task	54%	83%	See above			
**Affiliation and attachment (R: 3.74, P: 4.19)**						
Faux Pas test	74%	78%	See above			
Strange Stories Test	65%	74%	See above			
Reading the Mind in the Eyes Test (RMET or Eyes Test) (Baron-Cohen et al., 2001)^*^	61%	74%	Participants infer emotions and thoughts from images of eyes, gauging social cognition.	4	16+	Osterhaus and Bosacki (2022) • Internal consistency: reported by 6 studies (0.41, 0.53, 0.61, 0.62, 0.75. 0.82; M = 0.62, SD = 0.15)
**Frustrative non-reward (R: 4.13, P: 3.67)**						
Iowa Gambling Task (IGT) (Bechara, 2016)^*^	78%	83%	Assesses decision making through a card game where participants choose cards, learning to avoid risky options for long-term gains.	15-20 minutes to administer and score	8-79	• Split-half and test-retest: not testable (Lezak, 2012) • Low correlations with self-reported risk taking and personality traits related to risk-taking (Schmitz et al., 2020)
**Social communication (R: 4.00, P: 3.86)**						
Emotion Recognition Task (ERT)	78%	83%	See above			
Facial Expressions of Emotion – Stimuli and Tests (FEEST) (Young et al., 2002)^*^	78%	78%	Inferring emotions and thoughts from images of eyes, testing emotional perception and recognition. The FEEST is a combination of the Ekman 60 Faces Test and the Emotion Hexagon Test.	25-30	18+	Short version (Kuhlmann and Margraf, 2023) • Cronbach's α was on average 0.70 for prototype and 0.67 for morphed stimuli • Test-retest reliability: 0.60 for prototype and 0.62 for morphed stimuli Young et al. (2002) • Ekman 60 faces – split half: 0.62 (total score), 0.62 (anger), 0.66 (disgust), 0.53 (fear), 0.21 (happiness), 0.60 (sadness), 0.61 (surprise) • Emotion Hexagon – split half: 0.92 (total score), 0.68 (anger), 0.92 (disgust), 0.88 (fear), 0.18 (happiness), 0.65 (sadness), 0.33 (surprise) • Correlation between Ekman 60 faces and Emotion Hexagon: 0.68 (total score), 0.51 (anger), 0.27 (fear), 0.52 (disgust), −0.05 (happiness), 0.54 (sadness), 0.42 (surprise)
Faux pas test	74%	83%	See above			
Reading the Mind in the Eyes Test (RMET or Eyes Test)	74%	74%	See above			
**Sustained threat (R: 4.12, P: 3.68)**						
Reading the Mind in the Eyes Test (RMET or Eyes Test)	43%	52%	See above			
**Potential threat (“Anxiety”; R: 4.31, P: 3.44)**						
Emotion Recognition Task (ERT)	87%	91%	See above			
Affective/Emotional Go/No-Go Task^*^	78%	83%	Participants respond to emotional and neutral stimuli, measuring impulse regulation and emotional control.	Varies	Varies	Correlations between commission errors across the emotional and non-emotional tasks: 0.51-0.56, supporting the construct validity of behavioral inhibition (Schulz et al., 2007)
Facial Expressions of Emotion – Stimuli and Tests (FEEST)	65%	65%	See above			
**Acute Threat (“Fear”; R: 4.50, P: 3.24)**						
Emotion Recognition Task (ERT)	78%	83%	See above			
Affective/Emotional Go/No-Go Task	74%	78%	See above			
**Reward learning (R: 3.50, P: 3.76)**						
Iowa Gambling Task (IGT)	100%	100%	See above			
Wisconsin Card Sorting Task (WCST)	70%	87%	See above			
Tower of London (TOL)^*^	39%	78%	Participants move disks on pegs, aiming to recreate a specific tower arrangement, assessing strategic thinking, planning and problem solving.	10-15	7-80	Köstering et al. (2015) • Across samples, mean split-half and lower bound indices of reliability of accuracy scores were adequate (r ≥ 0.7) or higher, with the lower-bound estimate uniformly indicating high reliability (glb ≥ 0.8) • TOL-F planning accuracy possesses adequate criterion-related concurrent validity Humes et al. (1997) • Correlation with TOH: 0.37
Tower of Hanoi (TOH)^*^	39%	78%	Similar to TOL, except disks now vary in size (making the task more difficult)	15-20	7-80	Humes et al. (1997) • Correlation with TOL: 0.37 • Batista et al. (2007): Internal consistency 0.37 (original version) 0.40-0.77 (revised version)
**Attention (R: 3.65, P: 3.58)**						
D-KEFS: Trail Making Test (TMT) (Delis et al., 2001)	80%	88%	Connecting numbered circles while alternating between numbers and letters, evaluating cognitive flexibility, visual attention and attention shifting.	15-20	8-89	• Internal consistency 0.57 to 0.81 (Shunk et al., 2006); Low (≤ 0.59) (conditions 1-4) to Adequate (0.70–0.79) (condition 5) (Strauss et al., 2006) 0.72 (Combined Number + Letter Sequencing) (Delis et al., 2007c) Test-retest • Marginal (0.60–0.69) (Combined Number + Letter Sequencing) and Adequate (0.70–0.79) (motor speed and condition 5) (Strauss et al., 2006) 0.56 (cond. 1), 0.57 (cond. 2), 0.59 (cond. 3), 0.37 (cond. 4), 0.77 (cond. 5), 0.66 (combination) (Delis et al., 2007c)
WAIS: Digit Symbol Coding (Wechsler, 2012)	80%	92%	Matching symbols to numbers as quickly as possible, testing processing speed and sustained attention.	5	16-90	Test-retest • 0.86 (Pearson NL, 2012) • High (0.80–0.89) (Strauss et al., 2006) Internal reliability • High (0.80–0.89) (Strauss et al., 2006)
WAIS: Symbol Search (Wechsler, 2012)	80%	92%	Scanning sets of symbols, identifying target symbol presence or absence, assessing visual attention and processing speed.	5	16-90	Test-retest: • 0.75 (Pearson NL, 2012) • High (0.80–0.89) (Strauss et al., 2006) Internal reliability • Adequate (0.70–0.79) (Strauss et al., 2006)
**Loss (R: 3.72, P: 3.48)**						
Iowa Gambling Task (IGT)	42%	75%	See above			
Cambridge Gambling Task (CANTAB; (Cambridge Cognition, 2012)^*^	25%	50%	Participants choose between options to win or lose money, evaluating risk-taking, decision-making and reward-seeking behavior.	12-18	18+	Has been shown to be sensitive to impairment in gambling addition (Lawrence et al., 2009) and substance use disorder (Rogers, 1999).
**Reward Valuation (R: 3.18, P: 3.86)**						
Iowa Gambling Task (IGT)	96%	100%	See above			
Wisconsin Card Sorting Task (WCST)	57%	87%	See above			
**Reward Responsiveness (R: 3.26, P: 3.64)**						
Iowa Gambling Task (IGT)	96%	96%	See above			
Go/No-Go task	57%	65%	See above			
Stop Signal Task (SST) (Logan, 1994)^*^	43%	52%	Participants quickly respond to a visual or auditory signal but stop when a “stop” signal appears, assessing the ability to inhibit automatic responses.	Varies	Varies	Convergence with other types of self-control measures (Duckworth and Kern, 2011) • Executive functions: *r* = 0.11, N = 1982 • Delay tasks: *r* = 0.17, N = 189 • Self-report: *r* = 0.17, N = 402 • Informant-report: *r* = 0.13, N = 506
**Working Memory (R: 3.45, P: 3.37)**						
WAIS: Digit Span (Wechsler, 2012)	88%	92%	Participants repeat a series of numbers in the same order (forward) or reverse order (backward), assessing short-term/working memory capacity.	5	16-90	Test-retest: • 0.82 (Pearson NL, 2012) • Test-retest: High (0.80–0.89) (total), Adequate (0.70–0.79) (forward and backward) (Strauss et al., 2006) Split-half • 0.91 (Pearson NL, 2012) Internal reliability • High (0.80–0.89) (total, forward and backward) (Strauss et al., 2006)
WAIS: Letter Number Sequencing (Wechsler, 2012)	80%	88%	Participants listen to a sequence of numbers and letters, then repeat the numbers in ascending order followed by the letters in alphabetical order, evaluating working memory and attention.	5	16-90	Split-half • 0.81 (Pearson NL, 2012) Test-retest • 0.78 (Pearson NL, 2012) • High (0.80–0.89) (Strauss et al., 2006) Internal reliability • Very high (0.90+) (Strauss et al., 2006)
WMS-III: Spatial Span (Wechsler, 1997)	72%	88%	Participants recreate a sequence of blocks tapped by the examiner in the same order, testing visuospatial working memory.	5	16-90	Strauss et al. (2006) • Internal consistency: Adequate (0.70 to 0.79) • Generalizability coefficients: Adequate (0.70 to 0.79) • Test-retest: Adequate (0.70 to 0.79)
**Agency and Ownership (R: 3.48, P: 3.24)**						
Tower of London (TOL)	54%	79%	See above			
Tower of Hanoi (TOH)	50%	75%	See above			
D-KEFS: Tower Test (Delis et al., 2001)	42%	67%	measures executive functioning and planning abilities by assessing their capacity to rearrange a set of colored disks on pegs to match a target configuration while adhering to specific rules	15-20	8-89	Internal consistency • Marginal (0.60–0.69) (total achievement) (Strauss et al., 2006) 0.64 (Delis et al., 2007b) Test-retest • Low ( ≤ 0.59) (total achievement) (Strauss et al., 2006) • Test-retest: 0.44 (Delis et al., 2007b)
**Perception (R: 3.30, P: 3.23)**						
Rey Complex Figure Test (RCFT) (Meyers and Meyers, 1995)	68%	88%	Reproducing a complex figure from memory. Measures Visuospatial memory and organizational skills.	10-15 (excl. delays)	6-93	Strauss et al. (2006) • Test-retest: adequate to high for intervals of 6 months or less • Practice effects: yes
WAIS: Block Design (Wechsler, 2012)	56%	84%	Participants arrange blocks to match a given design as quickly as possible, assessing spatial reasoning, visual-motor skills. perceptual organization.	10-15	16-90	• Split-half: 0.84 (Pearson NL, 2012) • Internal reliability: High (0.80–0.89) (Strauss et al., 2006) • Test-retest: Adequate (0.70–0.79) to High (0.80–0.89) (Strauss et al., 2006)
Judgment of Line Orientation (JLO) (Benton et al., 1978)	52%	68%	Participants match lines in a diagram to angles in another diagram, evaluating spatial orientation and visual perception.	15-20	7-96	• Test-retest: high (Strauss et al., 2006)
**Sleep/Wakefulness (R: 3.34, P: 3.13): No tests were suggested**						
**Language (R: 3.19, P: 3.08)**						
Phonological fluency tests	83%	92%	Generating words starting with a specific letter	5	2-95 (depends on version)	Strauss et al. (2006) • Test-retest: adequate • Evidence of sensitivity to frontal damage: yes, but poor sensitivity and specificity • Evidence of ecological ability: yes
Semantic fluency tests	83%	92%	Generating words within a specific semantic category	5	2-95 (depends on version)	Strauss et al. (2006) • Test-retest: adequate • Evidence of sensitivity to frontal damage: yes, but poor sensitivity and specificity • Evidence of ecological ability: yes
WAIS: Comprehension (Wechsler, 2012)	83%	92%	Answering questions about social situations. Measures verbal comprehension and social knowledge	5-10	16-90	Pearson NL (2012) • Split-half: 0.84 • Test-retest: 0.78
**Circadian Rhythms (R: 3.26, P: 2.88): No tests were suggested**						
**Declarative Memory (R: 2.80, P: 3.13)**						
15 Words Test (15WT)/RAVLT	76%	80%	Recalling a list of words immediately after hearing. Measures verbal memory.	10-15	6-97	Strauss et al. (2006) • Test-retest: marginal to adequate for total recall, trail 5 and delayed recall trails • Practice effects: yes
Californian Verbal Learning Test (CVLT)	76%	76%	Memorizing and recalling a list of words over time. Measures verbal memory and learning over trials.	30 min, plus 30-min delay	16-90	Strauss et al. (2006) • Test-retest: high for scores of level of performance, low for scores of process/strategy • Practice effects: yes
Rey Complex Figure Test (RCFT)	68%	88%	See above			
**Motor Actions (R: 3.21, P: 2.72)**						
Go/No Go task	78%	83%	See above			
Trail Making Task (TMT)	65%	83%	Connecting numbers and letters in sequential order. Measures cognitive flexibility and visual attention.	5-10	9-89	Strauss et al. (2006) • Internal reliability: N/A • Test-retest: for the most part adequate • Evidence of sensitivity to attentional impairments: good • Evidence of ecological validity: good
Finger Tapping Test	61%	70%	Measures motor speed and coordination by tapping a finger as quickly as possible.	< 10	5-85	Strauss et al. (2006) • Test-retest: variable (low to high)
**Innate Motor Patterns (R: 2.69, P: 2.72)**						
Finger Tapping Test	43%	57%	See above			
Tower of Hanoi (TOH)	39%	61%	See above			
Tower of London (TOL)	39%	61%	See above			
**Habit - Sensorimotor (R: 2.81, P: 2.33)**						
WAIS: Digit Symbol Coding	50%	75%	See above			
Conner's Continuous Performance test (CCPT-II)^*^	46%	63%	Responding to specific target stimuli while ignoring distractions. Measures attention and impulse control.	14	6-55+	Strauss et al. (2006) • Internal reliability: acceptable to high • Test-retest: limited • Evidence of sensitivity to attentional impairments: moderate • Evidence of ecological validity: limited information
Stroop Test (Golden and Freshwater, 2002)	43%	70%	Naming the ink color of words while ignoring their meaning. Measures cognitive control and inhibition.	5	5-90 (depends on version)	Strauss et al. (2006) • Test-retest: adequate for the interference trial • Evidence of sensitivity to frontal damage: yes, but poor sensitivity and specificity • Evidence of ecological ability: yes

Note. ^*^ = digital version available; % suitable = percentage of the experts that rated the test as suitable to measure the given construct; % known = percentage of the experts that knew the test well enough to rate it's suitability. Only tests that were known by 50% of the panel were included in the table.

### 3.4 Positive valence systems

Positive Valence Systems were rated with M = 3.54 (SD = 0.11). Interestingly, this was the only domain that was deemed significantly more important for proactive (M = 3.76, SD = 0.11) than for reactive aggression (M = 3.32, SD = 0.14; *U* = 144, *p* < 0.001). The panel members reasoned that individuals with high reward responsiveness may be more motivated to engage in proactive aggression, driven by the pursuit of rewards and experiencing greater pleasure and motivation when such rewards are at stake. Reward learning is important for proactive aggression, as individuals who have learned that aggressive behavior leads to desired outcomes are more likely to repeat such behavior to achieve their goals. Lastly, the value of potential rewards shape proactive aggression, with those highly valuing rewards associated with aggression, like financial gain or social status, being more inclined to engage in this form of aggression. Reactive aggression is more indirectly related to reward as the alleviation of distress or protection can be considered the reward in this context.

The experts suggested 14 different tests to assess Positive Valence Systems. The top-3 tests are displayed in Table 3. For Reward Valuation, there were only two tests known by half of the panel. It was noted by the panel that many of these tests do not directly measure reactions to rewards, but this can be inferred through observation.

### 3.5 Arousal/regulatory systems

Arousal/Regulatory Systems were rated with M = 3.44 (SD = 0.50) importance overall, with non-significant difference between reactive (M = 3.66, SD = 0.63) and proactive (M = 3.21, SD = 0.38) aggression (*U* = 2, *p* = 0.400). The construct Arousal was considered very important in reactive aggression (M = 4.38), where impulsive and emotionally charged responses are common. Conversely, in proactive aggression (M = 3.63), the issue often revolves around the absence of arousal or under-arousal, suggesting a potential opposite relationship. It was highlighted that arousal is a state rather than a trait and is subject to rapid fluctuations influenced by environmental factors that can be challenging to measure. Disturbances in sleep and circadian rhythms could have consequences on daily mood patterns, possibly affecting emotional regulation and impulsivity.

The panel noted the absence of tests to measure arousal. Instead, they proposed physiological measures (such as EEG, heart rate variability, skin conductance, pupil dilation) behavioral/observational methods (such as wearables, questionnaires, or diaries), and neuroimaging. The seven tests that were suggested by the panel are often developed to measure different constructs such as motor skills, attention, and inhibition, and were all—except for the go/no-go task—unknown by half of the panel. Therefore, no top-3 could be validly constructed.

### 3.6 Sensorimotor systems

The domain with the lowest importance rating was Sensorimotor Systems (M = 3.18, SD = 0.48), with non-significant difference between reactive (M = 3.25, SD = 0.48) and proactive aggression (M = 3.10, SD = 0.51; U = 45, *p* = 739). The construct “sensorimotor integration” was suggested as an addition to this domain. The panel reasoned that sensorimotor systems might be relevant in understanding reactive aggression, which can be impulsive and driven by limbic responses, particularly in individuals with trauma or dissociation. These motor reactions can lead to a loss of agency and ownership over actions, potentially becoming self-fulfilling. Automatic aggressive behaviors learned from early experiences may be tied to sensorimotor patterns, particularly in reactive aggression. However, there's debate over whether these constructs can be clinically measured and if they directly correlate with quantifiable aggression.

Nevertheless, the panel suggested 39 different tests to measure Sensorimotor Systems. These tests encompass a wide range from executive functioning/planning tests (e.g., tower tests) to tests that more directly measure motor skills and coordination. A panel member proposed the idea of using advanced technology like movement sensors and virtual reality to understand how people physically react to challenging situations.

### 3.7 Additional suggestions

Lastly, the panel suggested four constructs that do not fit within the RDoC domains but might be worth considering when assessing aggressive offenders. For intelligence, the panel agreed (IQ*r* = 1) that this is moderately to very important to include (intelligence: reactive 3.54, proactive: 3.75). It was noted that general intelligence might not provide additional information beyond the specific cognitive functions already encompassed within the model or if these specific functions might completely explain the association between intelligence and aggression. Secondly, cognitive distortions—which are biased or irrational patterns of thoughts and perception that can influence a person's beliefs, attitudes, and behaviors—were deemed moderately to very important (IQ*r* = 1, reactive: 3.78, proactive: 3.87). A panel member noted that cognitive distortions are influenced by inner psychological patterns or past traumas and can cause a person to misinterpret what's going on, making them more likely to engage in violent behavior. Third, emotion regulation was rated as essential for reactive aggression (4.39, IQ*r* = 1), but there was no consensus for proactive aggression (3.74, IQ*r* = 2). Lastly, symptom/performance validity was added as a suggestion, but the panel did not reach consensus on this construct (reactive: 2.90, proactive: 3.14, IQ*r* = 2). The panel members commented that the addition of symptom/performance validity tests is valuable for detecting feigned or exaggerated symptoms and can help to ensure that decision about risk assessment/management and legal decisions are based accurate information. However, these type of tests are less directly related to understanding the origins of aggressive/offending behavior *per se*.

## 4 Discussion

In this Delphi study, we investigated two questions by surveying an international expert panel. Firstly, we sought theoretical insights into the constructs commonly associated with aggression, emphasizing their importance in the evaluation of predominantly reactive vs. predominantly proactive aggressive offenders. Secondly, we aimed to pinpoint the most suitable tests for this assessment, thereby facilitating future test selection in forensic contexts.

### 4.1 RDoC constructs

Overall, all RDoC domains were considered at least moderately important (>3) by the expert panel for the neuropsychological assessment of aggressive offenders. Taken together, Social Processes and Negative Valence Systems were rated as the most important in understanding aggression, while Sensorimotor Systems were considered least important. These findings are in line with studies that found a relation between aggression and executive functions and attention (Bergvall et al., 2001; Ogilvie et al., 2011; Burgess, 2020; Cruz et al., 2020), language (Cohen et al., 2003; Anderson et al., 2016; Chow et al., 2022), social cognition (Karoglu et al., 2022), and reward and threat processing (Estrada et al., 2019; Manning, 2020). Below, we will further explore the importance of the RDoC constructs considering the distinction between reactive and proactive aggression.

### 4.2 Reactive vs. proactive aggression

The extent to which experts differed in their opinion about the theoretical importance of the RDoC constructs for understanding reactive aggression compared to proactive aggression was rather small for most domains. The most pronounced difference was that Positive Valence Systems were deemed more important to understand proactive aggression, whereas Negative Valence Systems were considered most relevant for understanding reactive aggression. Both come as no surprise based on previous research. Differences in reward processing are found in children and adults with conduct disorder, callous unemotional treats, antisocial personality disorder and psychopathy (Estrada et al., 2019). As these diagnoses are generally related to proactive aggression (Merk et al., 2005; Cima and Raine, 2009; White et al., 2015; Zhang et al., 2017), this outcome fits well into what we know. In addition, studies have shown that in people with impulsive-antisocial traits linked to psychopathy, their brains released more dopamine in the nucleus accumbens when exposed to rewards, suggesting an hyperreactivity to rewards (Buckholtz et al., 2010). This highlights the relevance of trying to unravel the antecedents of aggression for assessment and treatment. Reactive aggression on the other hand is a primary reaction to perceived treat.

For the other domains, the difference in perceived importance between reactive and proactive aggression were rather small. This may be explained by the fact that some RDoC constructs, such as arousal, are quite broad. It has been reported in empirical studies that reactive aggression involves high affective-physiological arousal while proactive aggression is characterized by minimal autonomic arousal (Chase et al., 2001; Blair, 2003). In other words, arousal might be important in both types of aggression, albeit in different ways. Another example: compromised working memory might be associated with increased reactive aggression, as it is needed to process and react to triggers, while in proactive aggression, working memory is required for planning acts of violence, making it equally important but in a different manner. In other words, while RDoC constructs are important to evaluate to gain insights into the determinants of both forms of aggression, they may play different roles in the two types of aggression.

### 4.3 Expert recommendations for neuropsychological test usage

In total, 223 different tests were suggested by the panel. This indicates that an large number of neuropsychological tests have been developed in the past decades and attests to the field's rapid development. It also presents a challenge for clinicians in choosing the most suitable tests. In addition, aggression is a multifaceted construct that cannot be measured through a single test. The distinction between reactive and proactive aggression adds another layer of complexity. In response to these challenges, we constructed a guide for clinicians and researchers, a curated selection of the three most favored tests as assessed by our panel of experts.

It must be noted that our aim was to provide an overview that offers a selection of the most suitable tests to measure the RDoC constructs, rather than constructing a fixed battery of tests. By presenting an overview of the most important neuropsychological constructs along with the most suitable tests to measure them, clinicians and researchers can select specific constructs that are most relevant to their case. However, for certain subgroups, particularly when assessing patients with intellectual disabilities or patients who are illiterate, the tests suggested in our study might not be suitable. In those cases, clinicians are encouraged to seek for alternative tests. In the case of intellectual disabilities, it is proposed to use adapted versions of the original tests (such as the children's version) (Willner et al., 2010). In the case of illiteracy, the suggestion is to modify tests to resemble real-life situations instead of school-based procedures (Kosmidis, 2018). It is noteworthy that both intellectual disabilities and illiteracy more prevalent in forensic populations than in the general population (Harris, 2006; Tuominen et al., 2014; Hellenbach et al., 2017; Muñoz García-Largo et al., 2020), underscoring the importance of considering these factors in the selection of appropriate assessment tools. In addition, it is important to note that some of the tests that emerged from our study are subject to criticism, often in absence of better alternatives (e.g., the Thematic Apperception Test, see Lilienfeld et al., 2000). It is beyond the scope of this study to address tests individually.

Assessing these constructs may help to explain the determinants of the aggressive behavior which can provide valuable input for tailored treatment planning. Another important outcome is that the panel indicated that not every RDoC construct is appropriate to be measured by neuropsychological testing. For example, it was noted that the construct affiliation/attachment can be more effectively assessed through a structured interview, and arousal through observation or physiological measures. The RDoC matrix provides numerous examples of self-report and physiological measures for assessing its constructs (National Institute of Mental Health, 2023). Hence, a combination of neuropsychological tests, interviews, self-report, observation, and physiological measures might be needed to optimally measure the RDoC constructs.

### 4.4 Limitations

The findings of this Delphi study need to be considered in the context of a few limitations. Firstly, despite repeated and extensive attempts to include a representative global panel, half of the panel consisted of people from the Netherlands. The continents of South America and Africa were not represented at all and other continents were underrepresented (especially taking the number of inhabitants into account). Since neuropsychological practices are affected by, for example, the country's health care system, legal framework, and cultural norms, this is likely to have influenced the results of the study (Kasten et al., 2021). This may have also limited generalizability as certain recommendations might be more tailored to the Netherlands.

Furthermore, while every effort was made to ensure conciseness of the questionnaires, it is essential to recognize that participant motivation can influence the quality and consistency of expert input in iterative research endeavors like the Delphi method. Eight panel members (25% of the original panel, of which three from the Netherlands, 2 USA, 1 Italy, 1 Sweden, 1 Australia) did not complete all three rounds. This may have had implications for representativeness of the panel as they might have had different perspectives than the remaining 24 experts. Fortunately, most information was gathered in round 1 where experts rated all RDoC constructs and provided their test suggestions.

The panel generated a large number (223) of neuropsychological tests that can be used to measure the RDoC constructs. The panel was unfamiliar with many of the tests (56% of the tests were unknown to more than half of the panel), which prevented them from forming an opinion about their suitability. As a consequence, we could not validly construct a top-3 for each RDoC construct. For these constructs, we refer readers to the RDoC matrix for other assessment suggestions (National Institute of Mental Health, 2023).

Other limitations stem from the Delphi methodology. The approach toward consensus may exclude different but possibly important perspectives of individual panel members. The results of a Delphi study represent the ratings with the most overlap between the panel members, but this is not necessarily the “objective truth”. Our study wasn't designed to uncover objective truths; instead, we aimed to identify best practices. Moreover, the Delphi procedure precludes direct contact between panel members to avoid group pressure toward conformity and possible effects of authority. However, a discussion can often lead to valuable insights. To address this, the panel members could read each other's comments and reasonings anonymously in round 2 and 3. This could help them in understanding the source of potential discrepancies in ratings and possibly change their opinion. We highlighted that they were not obliged to change their ratings if their opinion had not been changed.

### 4.5 Implications and future directions

While this study represents a significant step forward in the endeavor to achieve adequate neuropsychological assessment of aggressive offenders, it is essential to acknowledge that our understanding of the relationship between the RDoC domains and aggression remains complex. Studying the interrelations between the constructs might provide more insights into aggressive behavior. For example, a lack of attention might lead to misinterpretation of social cues and a compromised working memory can lead to difficulties in emotion regulation.

Another prominent challenge that emerges from our study is the validation of the neuropsychological tests proposed by the expert panel. Moreover, to ensure that neuropsychological assessments are meaningful and sensitive to the unique characteristics of aggressive offenders, the field should focus on collecting more appropriate normative data.

Furthermore, the possible incorporation of neuropsychological test findings into risk assessment and management should be studied more thoroughly. This approach aligns with the Risk-Need-Responsivity (RNR) model (Bonta and Andrews, 2023), a leading framework in the forensic field. The findings have two connections to the RNR model. First, previous studies have indicated the added value of including biopsychosocial factors for the prediction of recidivism (Aharoni et al., 2013, 2014; Haarsma et al., 2020; Zijlmans et al., 2021). This aligns with the 'Need' principle of the RNR model, which emphasizes the importance of targeting criminogenic needs that are associated with an individual's likelihood of reoffending. Second, beyond understanding the cognitive limitations associated with aggression, future research should explore how this knowledge can be translated into effective intervention strategies. Specifically, cognitive limitations may play a crucial role in an individual's responsiveness to treatment, adhering to the 'Responsivity' principle of the RNR model. One might expect that if offenders have attentional difficulties or memory problems, that will influence treatment effectiveness. Longitudinal studies can help to understand how changes in neuropsychological function are related to changes in aggression and recidivism, further strengthening the connection between the RNR principles and the incorporation of neuropsychological assessments in risk assessment and -management strategies.

### 4.6 Conclusion

This Delphi consensus study shed light on the role of the RDoC framework in understanding and assessing aggression in offenders. The experts' ratings underline the multidimensional nature of aggression, calling for a holistic approach when assessing and addressing aggression. Furthermore, distinguishing between reactive and proactive aggression provides useful insights into the mechanisms involved in aggressive behavior.

The extensive list of proposed neuropsychological tests, as well as the construction of a top-3 list for each construct, provide clinicians and researchers with a useful resource when it comes to selecting suitable tests. This overview allows for a flexible approach by tailoring assessments to specific clinical or research requirements. Furthermore, the acknowledgment that certain constructs may be better examined through interviews, observations, or physiological measures emphasizes the added value of a multimodal assessment strategy.

Future research should focus on test validation, normative data collecting, and the integration of neuropsychological findings into risk assessment and intervention as our understanding of the complex relationship between RDoC domains and aggression advances. Our Delphi consensus study not only enhances our comprehension of aggression in offenders through the application of the RDoC framework but also provides a comprehensive guide for clinicians and researchers in the selection of neuropsychological tests. The findings of this Delphi study offer a steppingstone for advancing the field of neuropsychological assessment in understanding and addressing aggressive behavior.

## Data availability statement

The original contributions presented in the study are included in the article/Supplementary material, further inquiries can be directed to the corresponding author.

## Ethics statement

The studies involving humans were approved by Ethics Review Board (ERB) of the University of Amsterdam. The studies were conducted in accordance with the local legislation and institutional requirements. The participants provided their written informed consent to participate in this study.

## Author contributions

JCH: Conceptualization, Formal analysis, Investigation, Project administration, Writing – original draft. JEH: Supervision, Writing – review and editing, Conceptualization. SH: Writing – review and editing, Conceptualization. TZ: Supervision, Writing – review and editing, Conceptualization. HG: Writing – review and editing, Supervision, Conceptualization.

## Forensic Neuropsychology Consortium Members

Hasanen Al-Taiar, Nadia Bolognini, Lisanne Breuer, Josanne van Dongen, William Garmoe, Beverly Griffor, Jochem Jansen, Frank Jonker, Mike van Kessel, Niki Kuin, Femke Kuipers, Gary de Man, Bernice Marcopulos, Jesse Meijers, Pieter Molleman, Milo Oudenhoven, Rajakumari Reddy, J. Rajeshwaran Jamuna, Cristina Scarpazza, Anna van der Schoot, Carmen Sergiou, Andrea Stracciari, Marijke Willems, Stefano Zago.
