# Supplementary material for: Neuropsychological assessment of aggressive offenders: a Delphi consensus study
Source: Front Psychol. 2024 Feb 23;15:1328839. doi: 10.3389/fpsyg.2024.1328839 (PMC10922935; doi:10.3389/fpsyg.2024.1328839)
Supplement: Supplementary file 2 [file Table_2.docx]

## Supplementary Table S2

*Panel-ratings of all RDoC (sub)constructs for the neuropsychological assessment of predominantly proactive aggressive offenders.*

|  | Round 1 | | | | |  | Round 2 | | | | |  | Round 3 | | | | |
| --- | --- | --- | --- | --- | --- | --- | --- | --- | --- | --- | --- | --- | --- | --- | --- | --- | --- |
| RDoC constructs | n | range | M | SD | IQR |  | n | range | M | SD | IQR |  | n | range | M | SD | IQR |
| **Cognitive Systems** |  |  |  |  |  |  |  |  |  |  |  |  |  |  |  |  |  |
| Attention | 30 | 1-5 | 3.70 | 1.21 | 2 |  | 24 | 1-5 | 3.58 | 0.88 | 1 |  |  |  |  |  |  |
| Perception | 29 | 1-5 | 3.07 | 1.36 | 2 |  | 22 | 1-4 | 3.23 | 0.87 | 1 |  |  |  |  |  |  |
| Visual | 23 | 2-5 | 3.35 | 0.83 | 1 |  |  |  |  |  |  |  |  |  |  |  |  |
| Auditory | 23 | 1-5 | 3.13 | 0.97 | 2 |  | 22 | 1-5 | 3.05 | 0.90 | 0 |  |  |  |  |  |  |
| Olfactory/Somatosensory/Multimodal | 22 | 1-4 | 2.86 | 0.89 | 1 |  | 23 | 1-4 | 2.70 | 0.88 | 1 |  |  |  |  |  |  |
| Declarative Memory | 30 | 1-5 | 3.20 | 1.27 | 2 |  | 24 | 1-5 | 3.13 | 0.85 | 1 |  |  |  |  |  |  |
| Language | 30 | 1-5 | 3.03 | 1.25 | 2 |  | 24 | 1-5 | 3.08 | 0.83 | 1 |  |  |  |  |  |  |
| Cognitive Control | 31 | 2-5 | 4.16 | 0.93 | 1 |  |  |  |  |  |  |  |  |  |  |  |  |
| Goal Selection. Updating. Representation, and Maintenance | 31 | 3-5 | 4.26 | 0.68 | 1 |  |  |  |  |  |  |  |  |  |  |  |  |
| Response Selection; Inhibition/Suppression | 31 | 3-5 | 4.16 | 0.86 | 2 |  | 25 | 3-5 | 4.28 | 0.54 | 1 |  |  |  |  |  |  |
| Performance Monitoring | 30 | 2-5 | 4.17 | 0.79 | 1 |  |  |  |  |  |  |  |  |  |  |  |  |
| Working Memory | 30 | 1-5 | 3.37 | 1.19 | 1 |  |  |  |  |  |  |  |  |  |  |  |  |
| Active maintenance | 26 | 1-5 | 3.73 | 1.12 | 2 |  | 24 | 2-5 | 3.67 | 0.70 | 1 |  |  |  |  |  |  |
| Flexible Updating | 27 | 2-5 | 3.89 | 0.89 | 2 |  | 24 | 1-5 | 3.75 | 0.90 | 0 |  |  |  |  |  |  |
| Capacity | 28 | 1-5 | 3.50 | 1.11 | 1 |  |  |  |  |  |  |  |  |  |  |  |  |
| Interference Control | 28 | 2-5 | 3.86 | 1.01 | 2 |  | 24 | 2-5 | 3.88 | 0.74 | 1 |  |  |  |  |  |  |
| Counterfactual reasoning* |  |  |  |  |  |  | 19 | 2-5 | 3.68 | 0.75 | 1 |  |  |  |  |  |  |
| Information processing speed* |  |  |  |  |  |  | 24 | 2-5 | 3.50 | 0.72 | 1 |  |  |  |  |  |  |
| **Arousal/Regulatory** |  |  |  |  |  |  |  |  |  |  |  |  |  |  |  |  |  |
| Arousal | 32 | 1-5 | 3.56 | 1.39 | 2 |  | 24 | 2-5 | 3.63 | 0.65 | 1 |  |  |  |  |  |  |
| Circadian Rhythms | 29 | 1-5 | 2.66 | 1.23 | 2 |  | 23 | 2-4 | 3.04 | 0.82 | 2 |  | 24 | 2-4 | 2.88 | 0.54 | 0 |
| Sleep-Wakefulness | 29 | 1-5 | 2.83 | 1.28 | 3 |  | 23 | 2-4 | 3.13 | 0.76 | 1 |  |  |  |  |  |  |
| **Negative Valence Systems** |  |  |  |  |  |  |  |  |  |  |  |  |  |  |  |  |  |
| Acute Threat “Fear” | 32 | 1-5 | 3.25 | 1.16 | 2 |  | 25 | 1-5 | 3.24 | 0.83 | 1 |  |  |  |  |  |  |
| Potential Threat “Anxiety” | 32 | 1-5 | 3.44 | 1.05 | 1 |  |  |  |  |  |  |  |  |  |  |  |  |
| Sustained Threat | 32 | 1-5 | 3.75 | 1.02 | 2 |  | 25 | 1-5 | 3.68 | 0.80 | 1 |  |  |  |  |  |  |
| Loss | 30 | 1-5 | 3.50 | 1.17 | 2 |  | 23 | 1-5 | 3.48 | 0.85 | 1 |  |  |  |  |  |  |
| Frustrative Nonreward | 32 | 1-5 | 3.84 | 0.99 | 2 |  | 25 | 1-5 | 3.76 | 0.72 | 0 |  |  |  |  |  |  |
| The ability to learn from one's own errors* |  |  |  |  |  |  | 24 | 2-4 | 3.63 | 0.58 | 1 |  |  |  |  |  |  |
| **Positive Valence Systems** |  |  |  |  |  |  |  |  |  |  |  |  |  |  |  |  |  |
| Reward Responsiveness | 31 | 1-5 | 3.74 | 1.03 | 2 |  | 22 | 2-4 | 3.64 | 0.58 | 1 |  |  |  |  |  |  |
| Reward Anticipation | 29 | 2-5 | 3.90 | 0.98 | 2 |  | 21 | 3-4 | 3.76 | 0.44 | 1 |  |  |  |  |  |  |
| Initial Response to Reward | 29 | 2-5 | 3.72 | 0.92 | 2 |  | 23 | 2-5 | 3.70 | 0.63 | 1 |  |  |  |  |  |  |
| Reward Satiation | 28 | 2-5 | 3.71 | 0.94 | 2 |  | 22 | 2-5 | 3.68 | 0.65 | 1 |  |  |  |  |  |  |
| Reward Learning | 31 | 1-5 | 3.81 | 1.08 | 2 |  | 21 | 3-5 | 3.76 | 0.54 | 1 |  |  |  |  |  |  |
| Probabilistic and Reinforcement Learning | 26 | 2-5 | 3.96 | 0.96 | 2 |  | 22 | 3-5 | 3.91 | 0.43 | 0 |  |  |  |  |  |  |
| Reward Prediction Error | 26 | 2-5 | 4.04 | 0.92 | 2 |  | 22 | 3-5 | 4.00 | 0.53 | 0 |  |  |  |  |  |  |
| Habit - PVS | 24 | 2-5 | 3.63 | 1.01 | 1 |  |  | - |  |  |  |  |  |  |  |  |  |
| Reward Valuation | 31 | 1-5 | 3.68 | 1.08 | 2 |  | 21 | 3-4 | 3.86 | 0.36 | 0 |  |  |  |  |  |  |
| Reward (probability) | 28 | 2-5 | 3.93 | 0.98 | 2 |  | 23 | 2-5 | 3.78 | 0.60 | 1 |  |  |  |  |  |  |
| Delay | 26 | 2-5 | 3.81 | 0.94 | 2 |  | 21 | 2-5 | 3.71 | 0.72 | 1 |  |  |  |  |  |  |
| Effort | 24 | 2-5 | 3.83 | 1.09 | 2 |  | 20 | 2-5 | 3.70 | 0.73 | 1 |  |  |  |  |  |  |
| **Sensorimotor** |  |  |  |  |  |  |  |  |  |  |  |  |  |  |  |  |  |
| Motor Actions | 28 | 1-5 | 2.79 | 1.37 | 2 |  | 20 | 2-5 | 2.95 | 0.83 | 2 |  | 22 | 2-4 | 2.72 | 0.55 | 1 |
| Action Planning and Selection | 24 | 2-6 | 4.00 | 1.14 | 2 |  | 22 | 2-5 | 3.45 | 0.80 | 1 |  |  |  |  |  |  |
| Sensorimotor Dynamics | 24 | 1-6 | 3.79 | 1.53 | 0 |  |  |  |  |  |  |  |  |  |  |  |  |
| Initiation | 24 | 1-6 | 3.71 | 1.40 | 1 |  |  |  |  |  |  |  |  |  |  |  |  |
| Execution | 24 | 1-6 | 4.04 | 1.43 | 1 |  |  |  |  |  |  |  |  |  |  |  |  |
| Inhibition and Termination | 24 | 2-6 | 4.04 | 1.12 | 1 |  |  |  |  |  |  |  |  |  |  |  |  |
| Agency and Ownership | 28 | 1-5 | 3.25 | 1.29 | 2 |  | 21 | 1-5 | 3.24 | 0.94 | 1 |  |  |  |  |  |  |
| Habit – Sensorimotor | 27 | 1-5 | 2.67 | 1.14 | 2 |  |  |  |  |  |  |  |  |  |  |  |  |
| Innate Motor Patterns | 27 | 1-4 | 2.33 | 1.04 | 2 |  | 19 | 2-5 | 2.68 | 0.89 | 2 |  | 20 | 1-4 | 2.60 | 0.75 | 1 |
| Sensorimotor integration* |  |  |  |  |  |  | 17 | 1-4 | 2.82 | 0.81 | 1 |  |  |  |  |  |  |
| **Social Processes** |  |  |  |  |  |  |  |  |  |  |  |  |  |  |  |  |  |
| Affiliation and Attachment | 31 | 2-5 | 4.19 | 0.83 | 1 |  |  |  |  |  |  |  |  |  |  |  |  |
| Social Communication | 31 | 1-5 | 3.81 | 1.05 | 2 |  | 21 | 3-5 | 3.86 | 0.48 | 1 |  |  |  |  |  |  |
| Reception of Facial Communication | 29 | 2-5 | 3.86 | 0.95 | 2 |  | 22 | 3-4 | 3.68 | 0.48 | 1 |  |  |  |  |  |  |
| Production of Facial Communication | 28 | 1-5 | 3.11 | 1.17 | 2 |  | 21 | 2-4 | 3.33 | 0.66 | 1 |  |  |  |  |  |  |
| Reception of Non-Facial Communication | 28 | 2-5 | 3.86 | 0.89 | 2 |  | 21 | 3-4 | 3.57 | 0.51 | 1 |  |  |  |  |  |  |
| Production of Non-Facial Communication | 28 | 1-5 | 3.39 | 1.20 | 1 |  |  |  |  |  |  |  |  |  |  |  |  |
| Perception and Understanding of Self | 31 | 2-5 | 4.19 | 0.75 | 1 |  |  |  |  |  |  |  |  |  |  |  |  |
| Agency | 29 | 1-5 | 3.79 | 1.01 | 2 |  | 21 | 3-5 | 3.86 | 0.57 | 1 |  |  |  |  |  |  |
| Self-knowledge | 29 | 1-5 | 3.59 | 1.05 | 1 |  |  |  |  |  |  |  |  |  |  |  |  |
| Perception and Understanding of Others | 31 | 3-5 | 4.35 | 0.66 | 1 |  |  |  |  |  |  |  |  |  |  |  |  |
| Animacy Perception | 27 | 1-5 | 3.41 | 1.12 | 1 |  |  |  |  |  |  |  |  |  |  |  |  |
| Action Perception | 29 | 1-5 | 3.55 | 1.06 | 1 |  |  |  |  |  |  |  |  |  |  |  |  |
| Understanding Mental States | 31 | 2-5 | 4.06 | 0.77 | 1 |  |  |  |  |  |  |  |  |  |  |  |  |
| Ability to correctly understand the authenticity of others emotions* |  |  |  |  |  |  | 19 | 2-5 | 3.79 | 0.71 | 1 |  |  |  |  |  |  |
| Ability to understand absurdities* |  |  |  |  |  |  | 19 | 1-4 | 3.11 | 1.05 | 2 |  | 22 | 1-4 | 2.91 | 0.61 | 0 |
| Emotional contagion* |  |  |  |  |  |  | 18 | 2-5 | 3.33 | 0.84 | 1 |  |  |  |  |  |  |
| Moral reasoning* |  |  |  |  |  |  | 21 | 1-5 | 3.90 | 1.00 | 1 |  |  |  |  |  |  |
| Sympathy* |  |  |  |  |  |  | 21 | 1-5 | 3.57 | 0.98 | 1 |  |  |  |  |  |  |

*Note.* Rating ranges from 1 = not important, 2 = slightly important, 3 = moderately important, 4 = very important, to 5 = essential; IQR = interquartile range; n = Number of panel members rating the construct in question. As panel member only rated the subconstructs in case they rated the main construct as ≥ 3, the subconstructs were not rated by all panel members.
* These constructs are not part of the RDoC, but were suggested as additions in round 1.
